# From on-call to on-site: the impact of 24-hour in-house neonatology on billing patterns and physician productivity

**DOI:** 10.1038/s41372-025-02530-8

**Published:** 2026-01-05

**Authors:** Lee Donohue, Satyan Lakshminrusimha

**Affiliations:** Department of Pediatrics, UC Davis Children’s Hospital, Sacramento, CA USA

**Keywords:** Health care economics, Paediatrics

## Introduction

Attending coverage in many neonatal intensive care units (NICUs) has recently shifted from a model where attending physicians conducted rounds before dedicating the rest of the day to academic activities and taking night calls from home to a model where attendings are present in the NICU 24/7 [1, 2]. This change has been driven by factors including increased patient acuity, reduced availability of residents, decreased NICU exposure during medical training, and state mandates.

There are many potential impacts of this change. It increases demand for physicians as more will be needed to cover the added in-hospital hours. These additional hours, particularly at less desirable times, including nights and weekends, may detrimentally impact morale. The effects on patients have been explored with mixed results [3–7]. Trainee education may also be impacted [8].

Physician productivity is measured by work relative value units (wRVUs) which are generated based on Current Procedural Terminology (CPT^®^) codes billed. Physician billing at our institution is conducted primarily through professional coders. However, some attending physicians enter their own charges via the charge capture screen in EPIC®, although these charges are reviewed and edited as needed by the professional billing and coding team. While there has been a small amount of training for physicians in billing and coding, all charges entered by physicians are subject to review by the coding team. Neonatal nurse practitioners (NNPs) and neonatal hospitalists bill for services that are not supervised or attended by the physicians. These charges are coded and billed by the professional billing and coding team. As these NNPs, neonatal hospitalists and attending neonatologists are all hired by the university (which owns the hospital), all wRVUs and collections are pooled and collected by the health system.

Professional billing in the NICU utilizes bundled, global daily charges. These daily charges are billed by the person directing the care for that 24-hour period, who is generally the neonatologist working during the day. There may be a small amount of additional wRVUs generated that are attributed to admissions that occur late in the day but before midnight and non-bundled procedures at night. When the attending is not in-house at night, the initial care codes for new admissions are billed on the subsequent day. When the attending is in house at night, for admissions occurring before midnight, the initial care code is billed on the day of admission, and a subsequent care code is billed on the following day. Therefore, we hypothesized that there would be a slight increase in wRVUs generated with the shift to 24-hour in-house neonatologists.

## Methods

We collected divisional wRVUs, collections, CPT^®^ code frequency, average daily census, number of admissions, average length of stay, number of deliveries, case mix index, and days of intubated assisted ventilation for 2 years prior to, and 3 years following, the 2021 transition to 24-hour in-house attendings in the 49-bed NICU at UC Davis. The *t*-test was used to compare normally distributed data and the Mann–Whitney U test was used for non-normally distributed data.

We follow the Clinical, Administrative, Research, Teaching, Service (CARTS) or “one-minus” model at our institution, which assigns FTE time to various requirements of an academic neonatologist to determine what proportion of each individual’s full-time equivalent (FTE) will be clinical FTE (cFTE) [9, 10]. When attending physicians took calls from home, night on-call hours received 0.25 credit (a 16-hour home call night was considered equal to 4 daytime in-house hours, Table 1). The two physicians on service covered alternate night calls. When attending physicians stayed in the hospital, night calls received 12.5% additional credit compared to day shifts (Table 1) as suggested by experts for “hazard” compensation [11, 12]. This method was utilized to calculate the additional cFTE required when transitioning to the in-house attending model.

**Table 1:** Suggested cFTE credit for on-call (from home) or in-house coverage for a level III or IV NICU.

Attending physician location	Frequency of phone calls per shift	Frequency of telemedicine calls per shift or need to review X-rays and results	Need to drive in for emergencies	Credit per hour of call in terms of daytime, weekday hours^a^
Home	0–1	0	Rare (<1/10)	0
Home	1–5	1–2	10–25% of shifts	0.2
Home	6–10	>2	>25% of shifts	0.25–0.3
Home and Hospital	Part of the shift in the hospital regularly		>25% of shifts during the period at home	0.3–1 (based on % shift in the hospital)
Hospital	n/a	n/a	n/a	1.1–1.5^b^

^a^If the call from home is 15 h and the credit given is 0.3, then each call gets the physician 5 h of credit towards total clinical hours that year.

^b^Higher credit is given for shift differential/hazard coverage.

## Results

**Fig. 1:**
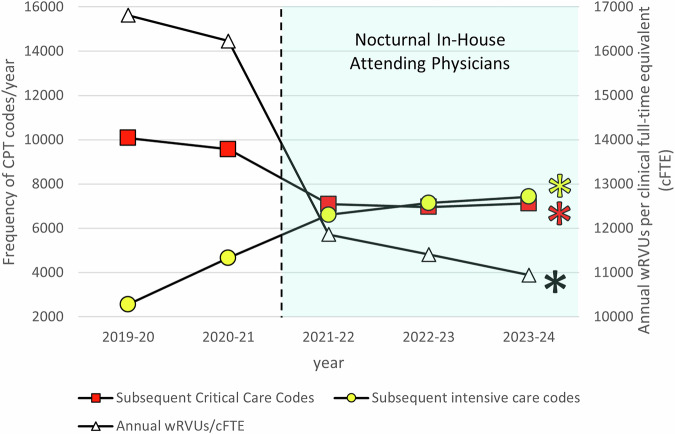
Trends in annual billing code frequency and wRVUs per cFTE from academic years 2019–20 through 2023–24. The left y-axis represents the frequency of billing codes per year, including subsequent critical care codes (red squares) and subsequent intensive care codes (yellow circles). The right y-axis represents annual wRVUs per cFTE (white triangles). The vertical dashed line indicates the time of implementation of nocturnal in-house attending physicians beginning in 2021–22, with the post-implementation period shaded in light blue. Asterisks on the rightmost data points indicate a statistically significant difference (*p* < 0.005) between the pre- and post-implementation eras.

The cFTE required to cover the NICU increased from 6.24 to 7.67 with the transition to an in-house staffing model, necessitating the hiring of two additional neonatologists. There was a 15% decrease in division wRVUs while the number of admissions and the average daily census were relatively unchanged (Table 2). The increase in cFTE and decrease in wRVUs resulted in a decrease in wRVUs/cFTE (Fig. 1). The decrease in wRVUs led to a decrease in collections (Table 2). The total wRVUs for the division include those generated by NNPs and neonatal hospitalists. These wRVUs are limited to procedures and delivery room attendance performed without a supervising physician and range from 164 to 233 wRVUs per year.

**Table 2:** Financial, staffing, and unit data before and after transition to in-house attending model in 2021.

	2019–2021	2021–2024	*p*
Divisional wRVUs, mean (SD)	103,090 (1812)	87,401 (926)	0.002
cFTE, mean (SD)	6.24 (0)	7.67 (0.29)	0.012
Years out of fellowship, median (IQR)	6.5 (4-21.5)	7 (5-9)	0.750
wRVUs/cFTE, mean (SD)	16,520 (290)	11,404 (373)	0.001
Collections^a^, mean (SD)	$6,998,438 (142,148)	$5,995,450 (287,078)	0.038
Collections per cFTE^a^, mean (SD)	$1,121,545 (22,780)	$781,294 (21,810)	0.001
Average daily census, mean (SD)	40.1 (1.7)	40.7 (0.9)	0.718
Admissions, mean (SD)	812 (100)	789 (22)	0.786
Average length of stay (days), mean (SD)	20.8 (2.8)	25.6 (1.9)	0.177
Deliveries, mean (SD)	1692 (102)	1874 (87)	0.196
Case mix index, mean (SD)	3.17 (0.26)	3.66 (0.10)	0.103
Subsequent IC codes billed, mean (SD)	3711 (1034)	7060 (340)	0.023
Subsequent CC codes billed, mean (SD)	9825 (252)	7057 (72)	0.001
Initial ICN codes billed, mean (SD)	166 (34)	266 (44)	0.123
Initial CC codes billed, mean (SD)	597 (62)	508 (52)	0.274
Days of assisted ventilation, mean (SD)	693 (69)	871 (139)	0.283
Patients with assisted ventilation, mean (SD)	50 (1)	65 (4)	0.024
Days of assisted ventilation per patient, mean (SD)	13.8 (1.1)	13.3 (1.4)	0.725

*IC* intensive care, *CC* critical care.

^a^Inflation-adjusted.

A closer examination of billing patterns revealed an increase in subsequent intensive care codes and a decrease in subsequent critical care CPT^®^ codes between the two eras (Fig. 1 and Table 2). The number of initial critical care codes decreased and initial intensive care codes increased but the difference was not statistically significant (Table 2).

The unit metrics were relatively unchanged over the study period. The number of admissions and average daily census (ADC) remained stable. The case mix index, a measure of patient acuity, increased although this was not statistically significant (Table 2). The number of days of intubated assisted ventilation and average number of days of intubated assisted ventilation per patient were unchanged.

Although there were some staffing changes during the study period, the median number of years out of fellowship was unchanged over the study period so the physicians had similar experience levels (Table 2).

## Discussion

There are relatively few CPT® codes used in neonatology, most of which are global codes covering care provided in a 24-hour period. These codes are based on the level of care required which is categorized as critical or intensive. Additionally, separate CPT® codes exist for initial and subsequent hospital care days. The wRVUs assigned to these levels of care are designed to reflect the time and complexity of patient management. The wRVU value for initial care codes is substantially higher than for subsequent care codes and the wRVU value of critical care codes is higher than that of intensive care codes [13].

Under the home call coverage model, attending physicians left the NICU after completing their work, with the remainder of the shift covered by fellows, residents and NNPs. The 24-hour presence of attending physicians provides up to an additional 15 hours/day of in-house coverage. The night attending physician performs several functions during this additional time in the NICU. They attend deliveries, direct transport calls, assist in patient care, and attend night rounds. Although they previously were involved in some of this from home, they are much more closely involved when they are present in the NICU.

While the responsibilities of the night attending are significant, the wRVUs generated are minimal. In a review of wRVUs generated by night attendings, we found that the night attending generates on average 14 wRVUs per night and some of these wRVUs would have been generated by the daytime attending physician in the home call model.

Contrary to our hypothesis, the total divisional wRVUs decreased with in-house attending physicians. During this period, the number of subsequent critical care codes decreased while the number of subsequent intensive care codes increased. The number of initial critical care codes was also decreased although not statistically significant. This shift in billing resulted in an associated decrease in collections.

One possible explanation for the shift in billing is that in-house attendings progress care such as weaning respiratory support more efficiently for patients in the NICU as well as attend more deliveries and are more involved in transports, resulting in improved patient stability. These changes may lead to a reduction in both initial and subsequent critical care coding.

This study has several limitations. First, the changes observed in CPT® codes and wRVUs were associated with the transition to 24-hour in-house attending coverage, but the study design does not establish a causal relationship. It was conducted retrospectively at a single center, which may limit generalizability to other NICUs. In addition, the reduction in both initial and subsequent critical care billing may have been due to an overall decrease in patient acuity, although the CMI numbers argue against this speculation. There was a trend towards a decrease in total wRVUs before implementation of 24-hour in-house coverage. Although we feel that this change was part of annual fluctuation, it is possible that some of the reduction in critical care codes was part of a process unrelated to in-house coverage. Finally, other unmeasured factors such as accuracy of billing and staffing changes may have influenced both clinical care and billing patterns.

While the transition to in-house attending physicians may enhance patient care, it also presents challenges. wRVU generation is used to determine staffing requirements and can impact divisional revenue and physician compensation. The decline in wRVUs despite stable workload in our analysis suggests that current wRVU-based metrics may undervalue physician effort in the in-house model. As institutions consider 24-hour staffing, it is important to account for these nuances in productivity metrics and finances when developing compensation models and staffing plans. Further research is needed to better understand the broader impact of this model on clinical outcomes and provider well-being.
